# ANS complex of St John’s wort PR-10 protein with 28 copies in the asymmetric unit: a fiendish combination of pseudosymmetry with tetartohedral twinning

**DOI:** 10.1107/S1399004715001388

**Published:** 2015-03-26

**Authors:** Joanna Sliwiak, Zbigniew Dauter, Marcin Kowiel, Airlie J. McCoy, Randy J. Read, Mariusz Jaskolski

**Affiliations:** a Center for Biocrystallographic Research, Institute of Bioorganic Chemistry, Polish Academy of Sciences, Poznan, Poland; b Synchrotron Radiation Research Section, National Cancer Institute, Argonne National Laboratory, Argonne, IL 60439, USA; c Department of Organic Chemistry, Poznan University of Medical Sciences, Poznan, Poland; d Department of Haematology, Cambridge Institute for Medical Research, University of Cambridge, Cambridge CB2 0XY, England; e Department of Crystallography, Faculty of Chemistry, A. Mickiewicz University, Poznan, Poland

**Keywords:** pathogenesis-related class 10 protein, St John’s wort, *Hypericum perforatum*, 8-anilino-1-naphthalene sulfonate

## Abstract

Hyp-1, a pathogenesis-related class 10 (PR-10) protein from St John’s wort (*Hypericum perforatum*), was crystallized in complex with the fluorescent probe 8-anilino-1-naphthalene sulfonate (ANS). The highly pseudosymmetric crystal has 28 unique protein molecules arranged in columns with sevenfold translational noncrystallographic symmetry (tNCS) along **c** and modulated X-ray diffraction with intensity crests at *l* = 7*n* and *l* = 7*n* ± 3. The translational NCS is combined with pseudotetragonal rotational NCS. The crystal was a perfect tetartohedral twin, although detection of twinning was severely hindered by the pseudosymmetry. The structure determined at 2.4 Å resolution reveals that the Hyp-1 molecules (packed as β-sheet dimers) have three novel ligand-binding sites (two internal and one in a surface pocket), which was confirmed by solution studies. In addition to 60 Hyp-1-docked ligands, there are 29 interstitial ANS molecules distributed in a pattern that violates the arrangement of the protein molecules and is likely to be the generator of the structural modulation. In particular, whenever the stacked Hyp-1 molecules are found closer together there is an ANS molecule bridging them.

## Abstract 2

Hyp-1, a pathogenesis-related class 10 (PR-10) protein from *H. perforatum*, was crystallized in complex with the fluorescent probe 8-anilino-1-naphthalene sulfonate (ANS). The asymmetric unit of the tetartohedrally twinned crystal contains 28 copies of the protein arranged in columns with noncrystallographic sevenfold translational symmetry and with additional pseudotetragonal rotational NCS.

## 1. Introduction   

The proteins that are expressed by plants under stressful conditions (such as drought, salinity or pathogen invasion), known as pathogenesis-related (PR) proteins, have been divided into 17 classes (Sels *et al.*, 2008 ▶). The members of most of these classes have well known biological activity. On this background, PR proteins of class 10 (PR-10) are very unusual because no unique function can be assigned to them despite their abundance, their coexistence as many isoforms in one plant, their differentially regulated expression levels and many years of study (Fernandes *et al.*, 2013 ▶). This is particularly surprising since the structure of PR-10 proteins has been very thoroughly studied and even defines a characteristic fold, also known as the Bet v 1 fold after the first protein from this class, a birch pollen allergen, to have its crystal structure determined (Gajhede *et al.*, 1996 ▶). The canonical PR-10 fold consists of an extended seven-stranded antiparallel β-sheet with a baseball-glove shape crossed by a long C-terminal helix α3, which is the most variable (in terms of both sequence and structural deformations) element of the PR-10 structure (Biesiadka *et al.*, 2002 ▶; Pasternak *et al.*, 2006 ▶). The seven β-strands form a consecutive progression connected by β-turns and loops, except for strands β1 and β2, which form the edges of the β-sheet and which are connected by a V-shaped motif of two α-helices (α1 and α2) that provides support for the C-terminal end of helix α3. The most intriguing feature of the PR-10 fold is the apparent lack of a proper hydrophobic core, in place of which there is a large hydrophobic cavity formed between the main structural elements, *i.e.* the β-sheet and helix α3. However, the hollow core does not lead to instability, as the PR-10 members are quite robust, resistant to proteases and have a mechanical stability that surpasses that of average globular proteins (Chwastyk *et al.*, 2014 ▶). The properties and the size of the internal cavity are modulated by the character of helix α3 in each particular case. The system of conserved β-bulges (which endow the β-sheet with its curvature) and numerous loops (L1–L9), some of which act as gating elements for the cavity, are also important for the PR-10 folding canon. The presence of the internal cavity naturally suggests a biological ligand-binding role. Indeed, several PR-10–ligand complexes have been characterized by crystallography, but their biological significance has only begun to emerge (Ruszkowski *et al.*, 2013 ▶, 2014 ▶). The persisting concerns are related to the fact that the physiological concentrations of phytohormones, which are the most frequently suggested ligands (Fernandes *et al.*, 2008 ▶; Ruszkowski *et al.*, 2014 ▶), are low compared with the binding constants, as well as to the observation that while the ligands in the crystal structures usually have excellent definition in electron density, they form diverse protein–ligand binding patterns. For example, similar or identical molecules are bound in multiple ways and even with variable stoichiometry. Additionally, complexes with PR-10 proteins are formed by phytohormones from totally divergent chemical classes, such as cytokinins (Pasternak *et al.*, 2006 ▶; Fernandes *et al.*, 2008 ▶), brassinosteroids or their analogues (Marković-Housley *et al.*, 2003 ▶), gibberellins (Ruszkowski *et al.*, 2014 ▶) and abscisic acid (Sheard & Zheng, 2009 ▶).

Direct determination of the binding constants, for example by isothermal titration calorimetry (ITC), is often difficult because of the low solubility displayed by most phytohormones. An alternative method, an ANS displacement assay, or ADA, is based on the fact that the fluorescent dye 8-anilino-1-naphthalene sulfonate (ANS) strongly changes its fluorescence in response to the chemical environment (Gasymov & Glasgow, 2007 ▶) and therefore can be titrated by another ligand that replaces it in a protein complex. ANS fluorescence is significantly increased after binding to a protein, with a hypsochromic shift of the fluorescence peak. To make full use of this method, the structural properties of ANS complexes with the target proteins should be well understood; as a minimum, the binding stoichiometry should be precisely known. Despite the popularity of the ADA method, it is surprising that there are only two deposited crystal structures (with coordinates) of the ANS anion [entries AMMANS (Weber & Tulinsky, 1980 ▶) and ANAPHS (Cody & Hazel, 1977 ▶)] in the Cambridge Structural Database (CSD; Allen, 2002 ▶) and that structural studies of ANS complexes with PR-10 proteins are scarce and limited to published structures of Bet v 1 complexes (PDB entries 4a80 and 4a8v; Kofler *et al.*, 2012 ▶) and an unpublished structure of a complex with a protein from the Andean crop jicama (PDB entry 1txc; F. Wu, Z. Wei, Z. Zhou & W. Gong, unpublished work). In the former case, the structure helped to explain the anomalous ANS fluorescence data at the molecular level. In the present study (first reported briefly in the context of molecular replacement with translational noncrystallographic symmetry; Sliwiak *et al.*, 2014 ▶), we have determined the crystal structure of an ANS complex of Hyp-1, a PR-10 protein from the medicinal herb St John’s wort (*Hypericum perforatum*). Hyp-1 was originally implicated (Bais *et al.*, 2003 ▶), most likely erroneously (Košuth *et al.*, 2013 ▶), as an enzyme catalyzing the biosynthesis of the pharmacological ingredient of the plant, the dianthrone hypericin, from two molecules of emodin. A subsequent crystallographic study of unliganded Hyp-1 demonstrated that the protein cavity (filled with serendipitous PEG molecules from the crystallization buffer) is indeed compatible with the size of one hypericin or two emodin molecules (PDB entry 3ie5; Michalska *et al.*, 2010 ▶). In this context, complex formation between Hyp-1 and ANS is of interest in itself as all of the implicated molecules (hypericin, emodin, ANS) contain large aromatic chromophores.

The Hyp-1–ANS complex studied in this work crystallized in a huge unit cell, with the basic motif of four protein molecules imperfectly repeated along **c**. Such translational noncrystallographic symmetry (tNCS) is sometimes called pseudotranslation. The presence of tNCS causes great difficulties in structure solution for two major reasons. Firstly, it can be difficult to work out how to break the exact lattice translational symmetry correctly. Secondly, most methods assume, at least implicitly, that the structure factors are all drawn from a uniform distribution, whereas in the presence of pseudotranslations there are extreme modulations in the intensity distribution, as seen here. In molecular replacement (MR) this can lead to false solutions because once one copy of a molecule has been placed (correctly or incorrectly), any copy placed in the same orientation but separated by the appropriate translation vector will reproduce the intensity modulation, thus improving the fit to the data without necessarily being correct. The maximum-likelihood methods for MR implemented in *Phaser* (McCoy *et al.*, 2007 ▶) depend on an accurate statistical model, so they were found to be highly sensitive to the failure to account for the statistical effects of tNCS. In order to solve the Hyp-1–ANS structure, it was necessary to adapt *Phaser* to account for these effects (Sliwiak *et al.*, 2014 ▶). Effectively, the entire set of molecules related by one or more translations is treated as a group, with the molecules rotating in concert during the rotation search and being translated as a group in the translation search. At the same time, the modulation of the error terms in the likelihood target is also accounted for.

To aggravate the problems even further, the crystal was found (belatedly, after the diffraction experiments had been finished) to be tetartohedrally twinned, which not only complicated the structure analysis as such but also resulted in an incomplete data set when indexed in the correct space group. However, in this case crystal twinning was actually used in a constructive way, *i.e.* to restore data completeness.

## 2. Materials and methods   

### 2.1. Protein preparation   

Hyp-1 was produced in *Escherichia coli* strain DE3 using the pET151/D vector with the *hyp-1* coding sequence and an N-terminal His-tag fusion (Fernandes *et al.*, 2008 ▶). 1 l LB medium was inoculated with 10 ml overnight culture grown at 310 K in the presence of 100 µg ml^−1^ ampicillin. At an OD_600_ of ∼1, the temperature was lowered to 291 K and isopropyl β-d-1-thiogalactopyranoside (IPTG) was added to a final concentration of 1 m*M*. After overnight culture, the cells were centrifuged at 6000*g* for 15 min at 277 K. The pellet was resuspended in lysis buffer [500 m*M* NaCl, 20 m*M* Tris–HCl pH 8.0, 20 m*M* imidazole, 3 m*M* β-mercaptoethanol, 100 µg ml^−1^ chicken egg-white lysozyme (Sigma–Aldrich)] and sonicated. The lysate was centrifuged at 18 000*g* for 15 min at 277 K. The supernatant was passed through a HisTrap column equilibrated with wash buffer (20 m*M* Tris–HCl pH 8.0, 20 m*M* imidazole, 3 m*M* β-mercaptoethanol) and eluted with 500 m*M* imidazole. The His tag was cleaved by His-tagged TEV protease with simultaneous dialysis against wash buffer at 277 K. After another round of affinity chromatography, the protein was purified on a size-exclusion column in 3 m*M* citrate buffer pH 6.3 with 150 m*M* NaCl. After purification, the protein was dialyzed against 3 m*M* citrate buffer and frozen at 193 K. The purified protein contains an N-terminal hexapeptide extension (GIDPFW–) as a cloning artifact. The final yield of recombinant Hyp-1 was 40 mg per litre of culture.

### 2.2. Complex formation, characterization and crystallization   

**Figure 1:**
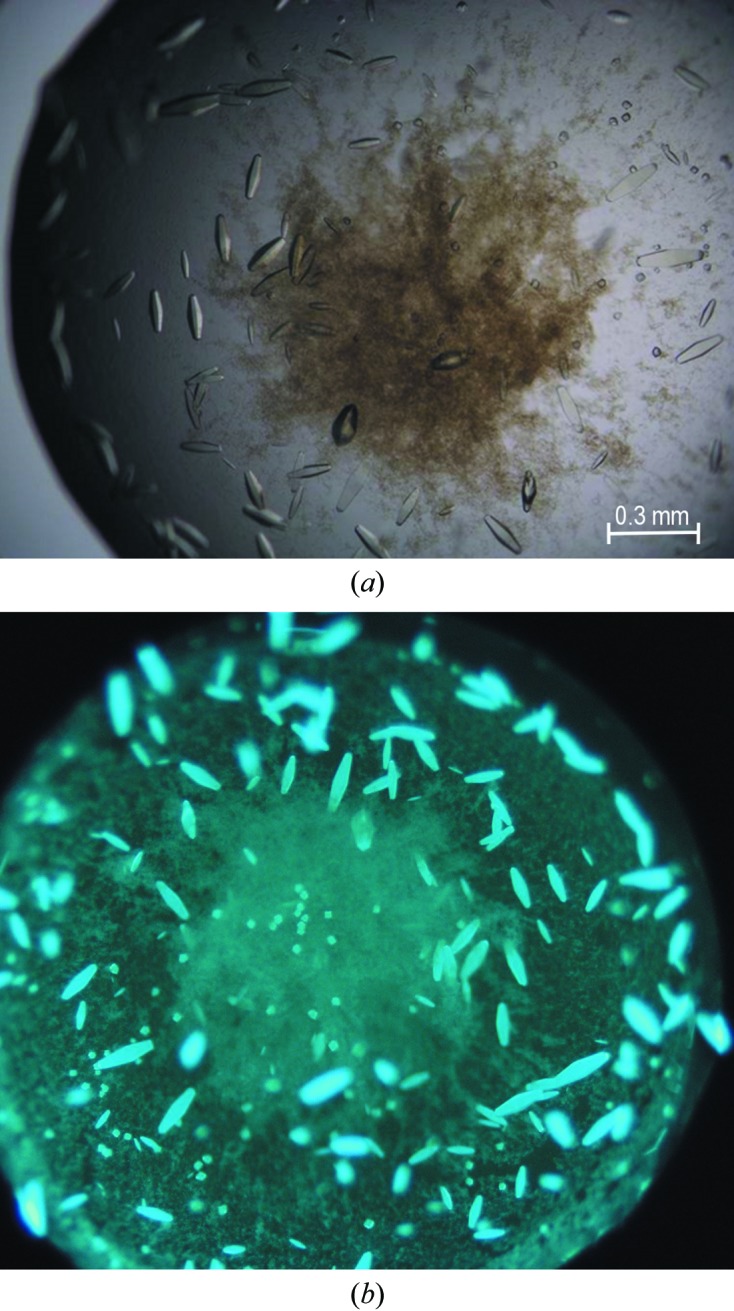
Single crystals of Hyp-1–ANS under a polarizing microscope (*a*) and a UV microscope (*b*).

For crystallization experiments, the protein solution was concentrated to 15 mg ml^−1^ and pre-incubated at 292 K for 1 h with an eightfold molar excess of ANS added from a 0.1 *M* stock solution in DMSO. Screening for crystallization conditions using Crystal Screen, PEG/Ion and PEG/Ion 2 (Hampton Research) was performed by the sitting-drop vapour-diffusion method against 120 µl well solution with the use of a Mosquito crystallization robot. The crystallization drops consisted of 0.2 µl protein–ligand solution and 0.2 µl well solution. Small crystals appeared after one week in 0.1 *M* HEPES pH 7.5 with 1.4 *M* tribasic sodium citrate as the precipitant. The preliminary crystals were used for seeding in a gradient of PEG 400 or glycerol and tribasic sodium citrate. Large crystals of dimensions 0.1 × 0.1 × 0.3 mm (Fig. 1 ▶ *a*) appeared in 0.1 *M* HEPES pH 7.5, 10% glycerol, 1.3 *M* tribasic sodium citrate. Strong blue fluorescence observed under a UV microscope (Fig. 1 ▶ *b*) confirmed the presence of ANS in the crystals.

### 2.3. X-ray diffraction data collection and processing   

**Figure 2:**
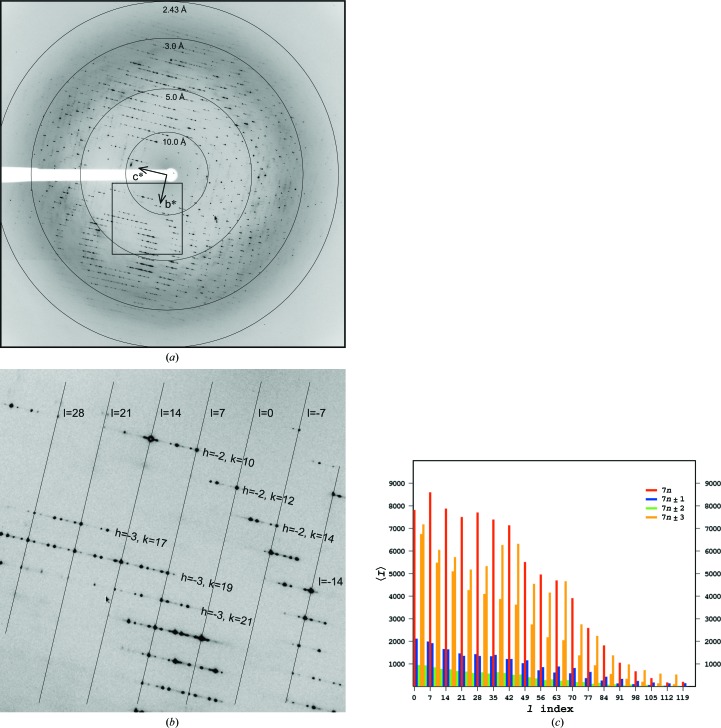
X-ray diffraction images of Hyp-1–ANS crystals. (*a*) A sample full image, with resolution rings and reciprocal-lattice directions indicated, and (*b*) an enlarged fragment on which layer lines of constant *l* = 7*n* are marked and annotated; the perpendicular layer lines have *h*, *k* indices as annotated. (*c*) A histogram of intensity distribution in layers of *l*.

Diffraction data collection and processing, including the treatment of data incompleteness resulting from the acceptance of apparent *P*422 crystal symmetry arising from perfect tetartohedral twinning and the eventual choice of *C*2 symmetry following molecular replacement in *P*1, have been described previously (Sliwiak *et al.*, 2014 ▶). The diffraction images recorded to 2.43 Å resolution revealed a repetitive sevenfold modulation (Fig. 2 ▶) of the reflection intensities along the longest lattice dimension (*c*), which was interpreted as an indication of a sevenfold noncrystallographic translation of a structural pattern along **c**.

As noted previously, the strategy adopted during data collection, adjusted for tetragonal symmetry, turned out to be inadequate for the *C*2 cell. The 90° of crystal rotation covering the asymmetric unit of the 422 symmetry corresponded to two equivalent 45° ranges instead of the full 90° wide monoclinic asymmetric unit, yielding only ∼73% data completeness. However, the presence of perfect tetartohedral twinning suggested an opportunity to expand the data from tetragonal to monoclinic symmetry without introducing significant errors, since in the case of perfect tetartohedry the data agree with the 422 symmetry anyway.

### 2.4. Structure solution   

The procedure that led to the solution of the crystal structure has been outlined before (Sliwiak *et al.*, 2014 ▶). Briefly, MR trials in all space groups consistent with a *P* lattice and point group 422 yielded multiple similar potential solutions in space group *P*4_1_22, but this symmetry was ruled out by strong 00*l* ≠ 4*n* reflections. Coupled with evidence of twinning, this suggested that the true symmetry was lower, but it was not clear which of the many potential subgroups of 422 point-group symmetry would be correct. Accordingly, structure solution was attempted in space group *P*1, searching for 56 copies of Hyp-1. Alhough one copy of the model comprises less than 2% of the scattering power, the search accounting for tNCS actually looked for seven copies at a time (in accord with strong native Patterson 0, 0, *w* peaks at *w* = *n*/7), making the problem tractable. This search succeeded in finding a unique solution, and the correct *C*2 symmetry was deduced by analyzing the symmetry of the calculated structure factors as described below. The MR solution in *C*2 symmetry was obtained by searching for four copies of the first set of seven molecules from the *P*1 solution.

### 2.5. Structure refinement   

About 3000 (1.3%) *R*_free_ reflections were selected in *SHELXPRO* (Sheldrick, 2008 ▶) in narrow resolution shells to ensure the inclusion of twin-related and NCS-related reflections. The structure was refined in *REFMAC*5 (Murshudov *et al.*, 2011 ▶) with an intensity-based twin-detection/refinement and jelly-body refinement mode. For the protein molecules, the standard stereochemical restraint library was used (Engh & Huber, 1991 ▶). The geometrical restraints for the ANS molecules were created using the coordinates of the magnesium salt of ANS (Cody & Hazel, 1977 ▶) found with reference code ANAPHS in the Cambridge Structural Database (Allen, 2002 ▶). Briefly, stereochemical targets from this structure were applied to covalent bonds, planar groups and three torsion angles, τ_1_ (O2—S—C9—C10), τ_2_ (C10—C1—N—C11) and τ_3_ (C1—N—C11—C16), with weights adjusted for bonds, planarity and torsions using 0.02 Å, 0.02 Å and 20°, respectively, as the standard deviations. Valence *sp* ^2^ angles were restrained at 120 (3)°. The refinement statistics are summarized in Table 1 ▶.

### 2.6. ANS binding assay   

Fluorescence measurements were carried out at room temperature using an RF-5301 Shimadzu spectrofluorimeter and the following conditions: λ_exc_ = 378 nm and λ_em_ = 470 nm with 5 nm excitation and emission slits. Concentrated protein (2.6 m*M*) was titrated in 4–50 µl aliquots into a cuvette containing 2.5 ml 1 µ*M* ANS solution in HEPES buffer (25 m*M* HEPES pH 7.4, 150 m*M* NaCl, 10 m*M* MgCl_2_, 1 m*M* β-mercaptoethanol). After each injection, the sample was mixed by pipetting. The fluorescence data (*F*) plotted against protein concentration were fitted using the equation *F* = *F*_max_[protein]/(*K*_d_ + [protein]), where *K*_d_ is the dissociation constant.

### 2.7. ITC measurements   

ITC titrations were carried out at 298 K using a MicroCal iTC200 calorimeter (GE Healthcare). Before the experiment, the protein was dialyzed against a buffer consisting of 150 m*M* NaCl, 25 m*M* HEPES pH 7.4, 1 m*M* β-mercaptoethanol. ANS was dissolved in the dialysis buffer to a concentration of 5 m*M*. The protein concentration in the sample cell (145 µ*M*) was determined by the Bradford assay (Bradford, 1976 ▶). The ligand solution was injected in 54 aliquots of 1.5 µl each until saturation was observed. The ITC data were analyzed with the *Origin* 7.0 software (OriginLab) to obtain the following parameters: stoichiometry (*N*), dissociation constant (*K*_d_) and the changes in enthalpy (Δ*H*) and entropy (Δ*S*) during the complexation reaction. The experimental curves were fitted using one set of binding sites as the model.

## 3. Results and discussion   

### 3.1. Treatment of diffraction data: transformation from tetragonal to triclinic to monoclinic symmetry   

The exploration of possible symmetries has been described previously (Sliwiak *et al.*, 2014 ▶), but the details of the statistics on which the decisions were based were not presented.

Because of the initial ambiguity in the true space group of the structure introduced by the physical twinning of the crystal, it was decided to solve the structure by MR in the triclinic *P*1 space group after expansion of the diffraction data to the Ewald hemisphere. The transformation (from *P*422 to *P*1) retains the unit cell but ignores its symmetry, *i.e.* it expands reflections in the same axial system and with the same indices.

After the structure had been solved in the *P*1 unit cell, the 56 copies of Hyp-1 were subjected to rigid-body refinement in *phenix.refine* (Afonine *et al.*, 2009 ▶). The symmetry of the MR solution was determined using *POINTLESS* (Evans, 2006 ▶) to analyze the relationships among *F*_calc_ structure amplitudes, which were evaluated in terms of correlation coefficients and merging *R* factors between reflections related by potential symmetry operations (Table 2 ▶ *a*). The agreement was excellent for only one symmetry element, a twofold axis oriented along one of the original tetragonal diagonals, which becomes the unique monoclinic **b** axis after reindexing. The second diagonal becomes the crystallographic **a** direction (without any symmetry), and this choice of axes creates the *C* centring. The original tetragonal **c** direction becomes the **c** axis of the monoclinic cell and loses its crystallographic symmetry. The asymmetric unit of the monoclinic lattice contains 28 protein molecules, labelled *A*, *B*, …, *Z*, *a*, *b*.

After the structure was re-solved in the *C*2 unit cell, it was again subjected to rigid-body refinement. This yielded the same *R* factor (43.2%) as the *P*1 solution, supporting the conclusion that the twofold axis was indeed crystallographic. Analysis with *POINTLESS* (Evans, 2006 ▶) showed that there was no further undetected symmetry in the calculated structure factors (Table 2 ▶ *b*). Although there is significantly better agreement between reflections related by the pseudo-twofold axis parallel to c* than for other potential symmetry operators, this operator could only be crystallographic if the other diagonal twofold, which gives much poorer agreement, were also crystallographic.

### 3.2. Data statistics and detection of twinning in the presence of translational pseudosymmetry   

The translational pseudosymmetry causes a modulation of the diffraction pattern in which the strongest intensities tend to have *l* indices near multiples of 3.5. Accordingly, the native Patterson map has strong peaks at 0, 0, *n*/7, with the strongest peak at *w* = 2/7. The modulation broadens the distribution of intensities, thereby masking the statistical effect of twinning. A complete analysis of the statistical effect of tNCS can unmask the effect of twinning on intensities (Read *et al.*, 2013 ▶), but the algorithm in *Phaser* is currently only able to model this with sufficient sophistication (including the differences in orientation of tNCS-related copies) in the case of twofold tNCS.

The *L*-test (Padilla & Yeates, 2003 ▶) provides an independent method to unmask the effect of twinning by looking at pairs of reflections separated by vectors in reciprocal space chosen to remove the correlation from tNCS. By default, the *L*-test uses pairs of reflections separated by multiples of 2 in *h, k* and *l*, at least in some implementations. In the present case, reflections separated by 2 in *l* are actually anticorrelated, because this is approximately half of the distance between peaks in the intensity distribution separated by 3.5 in *l*. This explains why, in standard applications, the test appears to suggest negative twinning! The *phenix.xtriage* program (Zwart *et al.*, 2005 ▶) tries to find a better default separation by taking the inverse of the nonzero coordinates of the top Patterson peak, but unfortunately in the present case the top peak at *w* = 2/7 yields 4 as the nearest integer. This gives a slightly more sensible, but still suboptimal, *L*-test result. To obtain an optimal *L*-test for this case, *phenix.xtriage* was run with a separation of multiples of 7 along *l*, using the expert option l_test_dhkl = ’2,2,7’. With this separation (and the default of multiples of 2 along *h* and *k*), the *L*-test gives 〈|*L*|〉 = 0.458 and 〈*L* ^2^〉 = 0.288 for the data merged in *P*422, indicating at least partial twinning. Note that twinning tests based on intensity distributions will underestimate the extent of twinning that parallels the pseudosymmetry, because the intensities of reflections related by pseudosymmetry will be correlated, thus reducing the perturbations in the intensity statistics introduced by twinning. In addition, there are no twin laws for 422 or pseudomerohedral twin laws for this cell in this symmetry, so a crystal cannot both have *P*422 symmetry and suffer from only partial twinning.

### 3.3. Structure refinement and model quality   

**Figure 3:**
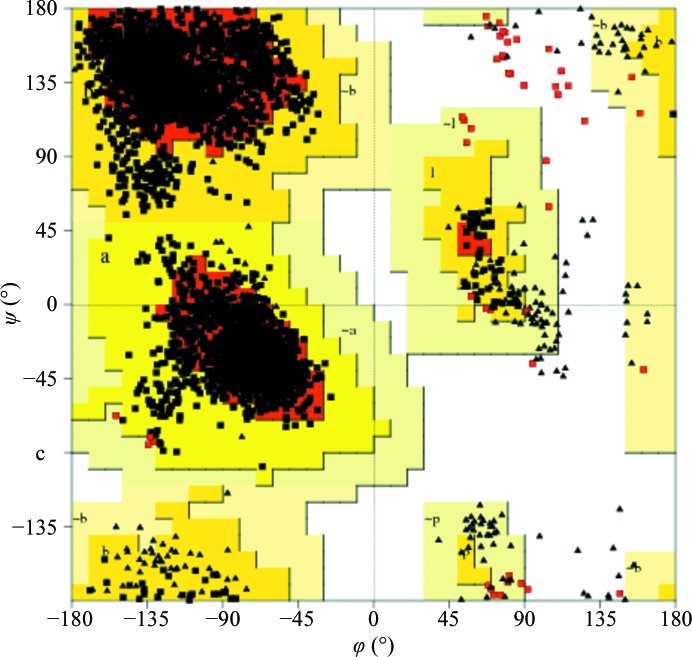
Ramachandran plot for the 28 copies of Hyp-1 in the asymmetric unit, generated by *PROCHECK* (Laskowski *et al.*, 1993 ▶). Gly residues are marked by triangles; residues in disallowed regions (22; 0.6%) are in red.

**Figure 4:**
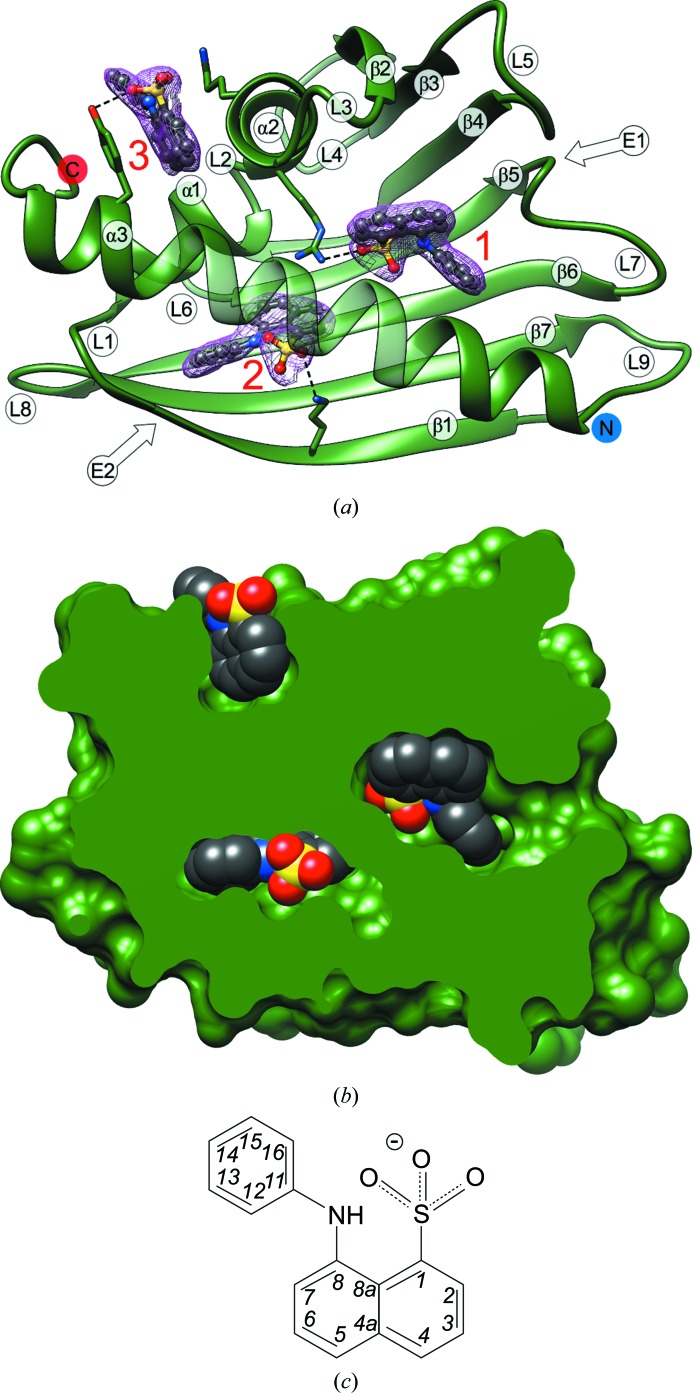
ANS binding sites 1, 2 and 3 in copy *L* of Hyp-1 (*a*) in relation to the (labelled) secondary-structure elements of the Hyp-1 fold, with ANS molecules in ball-and-stick representation, and (*b*) in van der Waals representation to emphasize the excellent fit to the shape of the binding pockets. The ANS molecules in (*a*) are shown in their 2*F*_o_ − *F*_c_ electron density contoured at 1.5σ. Dashed lines indicate hydrogen bonds to protein atoms. Additionally, Lys33 forms hydrogen bonds to ANS at site 3 in some copies of Hyp-1. (*c*) Covalent structure and IUPAC atom numbering of ANS (Jaskolski, 2013 ▶).

The final refinement converged with an *R* factor of 22.3%, yielding a model with very good stereochemical quality (Table 1 ▶). Analysis of the outliers in the Ramachandran plot (presented in Fig. 3 ▶ as a consolidated plot generated in *PROCHECK*; Laskowski *et al.*, 1993 ▶) carried out in *MolProbity* (Chen *et al.*, 2010 ▶) suggests that violations of main-chain conformation are found mainly in four loop areas, L4, L7, L8 and L9, which are usually well defined in other PR-10 structures. Conversely, the C-terminal helix α3, which is often disordered, especially in ligand-free PR-10 structures, is well ordered in Hyp-1. The final electron-density maps allow the tracing of all 28 Hyp-1 chains without gaps. Also, most of the side chains have very clear definition. The high quality of the electron density is illustrated by the fact that 89 copies of the ANS molecule could be confidently modelled in difference electron-density maps phased by the protein component only immediately after MR (Fig. 4 ▶ *a*). 60 of the ANS ligands are tightly docked (Fig. 4 ▶ *b*) within three binding sites (denoted 1, 2 and 3) of Hyp-1, but the ligand saturation is not complete (*i.e.* there are fewer than 28 × 3 = 84 docked ligands). However, one can easily identify protein chains that are totally empty (*T* and *V*) or have one or two binding sites occupied, as well as the 11 copies of Hyp-1 that are fully saturated with three docked ligand molecules. Moreover, an additional 29 ANS molecules with very good electron density were found at selective sites between Hyp-1 molecules. Their locations can be grouped into five superficial sites (denoted 4–8). Surprisingly, despite the huge asymmetric unit cell, only 35 water molecules could be confidently identified in the structure.

### 3.4. Hyp-1–ANS as a modulated superstructure   

The appearance of the diffraction pattern, with an alternation of strong (main) and weak (satellite) reflections in the c* direction (Fig. 2 ▶), and the appearance of the crystal packing in direct space, with a sevenfold translational repetition of the same structural pattern (two Hyp-1 dimers related by ∼180° rotation and ∼1/14 translation) in the **c** direction, both suggest that this is a case of a modulated superstructure (Wagner & Schönleber, 2009 ▶). However, the successful indexing of the diffraction pattern (of both the main and satellite reflections) with a simple three-dimensional lattice, in which the satellites divide the distances between the main reflections in a rational manner, indicates that the modulation is commensurate. It is thus possible to simplify the description of the structure using an expanded (sevenfold in the **c** direction) supercell, without resorting to the more rigorous but also much more complicated description in superspace that would be necessary in an incommensurate case.

### 3.5. Crystal packing of the protein molecules   

#### 3.5.1. Dimerization of Hyp-1   

**Figure 5:**
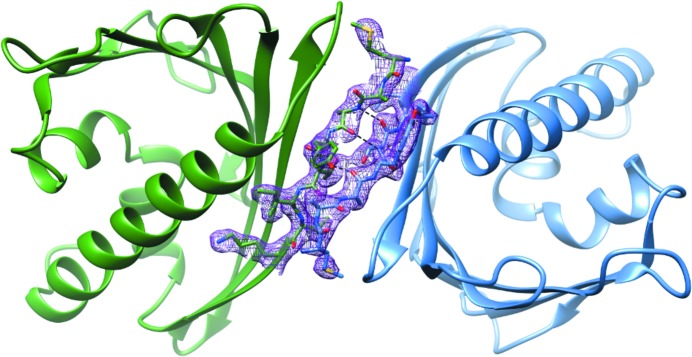
Hyp-1 dimer formation in the crystal structure shown in 2*F*_o_ − *F*_c_ electron density (contoured at 1.5σ) for protein molecules *K* and *L*. The antiparallel intermolecular β-sheet is created by the β1 strands from both molecules.

**Figure 6:**
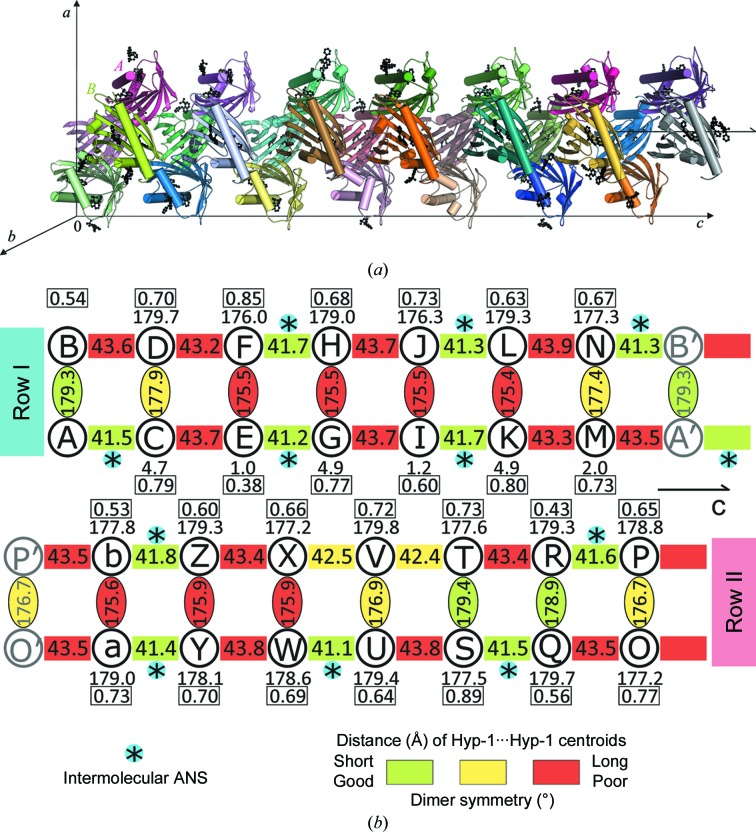
(*a*) The 28 independent Hyp-1 molecules forming the asymmetric unit of the crystal packing (with molecules *A* and *B* labelled), arranged in a dimeric pattern with a sevenfold repeat around a noncrystallographic 2_1_ screw axis along the crystallographic **c** direction. The ANS ligands are shown as black ball-and-stick models. (*b*) Arrangement of Hyp-1 dimers (labelled by protein chain identifiers *A*, *B*…, *b*) in two rows (*AB*, …, *MN* and *ab*, …, *OP*) along **c**. Protein copies translated along **c** are marked with a prime. Centroid distances between consecutive Hyp-1 molecules are marked in Å. Cross-linking of consecutive Hyp-1 pairs through an interstitial ANS molecule is marked by *. NCS symmetry of the dimers is indicated by the degree of rotation between the two chains. The rotation required for the best superposition of molecule *A* onto the remaining Hyp-1 C^α^ traces is given for each chain, with the corresponding r.m.s.d. (in Å) boxed. All rotations (in °) were calculated in *ALIGN* (Cohen, 1997 ▶).

In keeping with the majority of other PR-10 proteins, Hyp-1 is monomeric in solution, as confirmed by size-exclusion chromatography and native PAGE electrophoresis (not shown), and is expected to be biologically relevant as a monomer. Nevertheless, we note that there is a precedent of functional dimerization of a PR-10 protein (*Medicago truncatula* MtN13; Ruszkowski *et al.*, 2013 ▶) and that in the previously reported unliganded Hyp-1 structure (with PEG molecules found in the protein cavity) the protein molecules were linked into dimers *via* an S—S bond between cysteine residues Cys126 (quite rare in PR-10 sequences). In addition, in another crystal-packing contact two Hyp-1 molecules formed an intermolecular β-sheet through parallel association of their β1 strands. It is interesting to note that in the present structure all of the multiple copies of Hyp-1 are also paired into dimers *via* intermolecular β1–β1 inter­actions. At variance with the previous structure, all of the present β1–β1 sheets are antiparallel, thus flawlessly extending the intramolecular β-sheet from one molecule to the other (Fig. 5 ▶). In the adopted labelling scheme, the Hyp-1 dimers are *AB*, *CD*, …, *ab*. Seven of these dimers (*AB*, …, *MN*) have the same orientation and similar, repetitive spacing along the **c** axis, forming a discernible row (denoted I) in this direction. The remaining seven dimers are copies of the former dimers through a noncrystallographic 2_1_ screw axis along **c** and form another row (II) in this direction. In effect, this zigzag packing arrangement follows a noncrystallographic 2_1/7_ screw axis with ∼180° rotation and ∼1/14 translation (Fig. 6 ▶). The 29 interstitial ANS molecules have a similar but not identical disposition with respect to the sevenfold symmetric packing of the protein molecules. This deviation from perfection explains why the crystal has a unit cell with pseudo-sevenfold translation along the **c** axis.

#### 3.5.2. Higher-order association in the crystal lattice   

**Figure 7:**
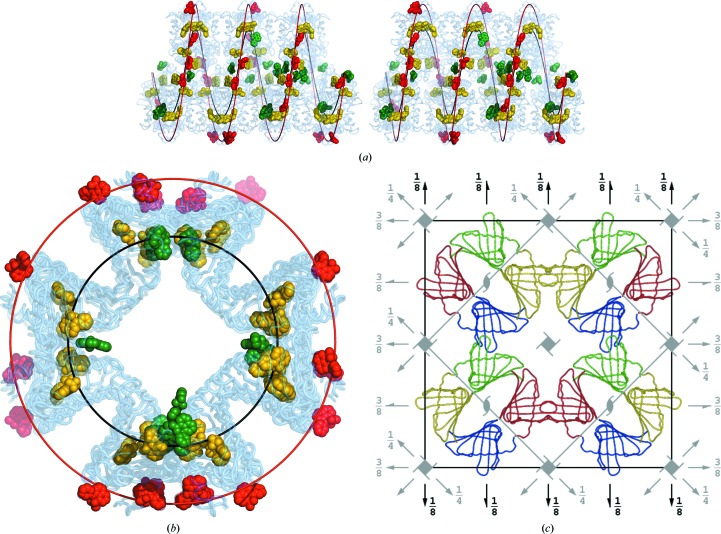
(*a*, *b*) Interpretation of the structure in terms of helical motifs. The black helical line was fitted to the centres of the consecutive *ba*–*NM*–*AB*–*OP*–*ZY*–*LK*–*CD*–*QR*–*XW*–*JI*–*EF*–*ST*–*VU*–*HG* dimers with 0.0 ≤ *z* < 0.5. The ANS molecules at binding sites 1 and 2 are in yellow, those at binding sites 3, 4, 5 and 6 are in red and those at binding sites 7 and 8 are in green. The red helical line was fitted to the ANS molecules marked in red. Views down the **b** (stereo) (*a*) and **c** (*b*) axes are shown. (*c*) The *C*2 unit cell and its symmetry elements (black) and the approximate *P*4_1_22 unit cell and its symmetry elements (grey). The four rows in each Hyp-1 column are shown in different colour (red/green and blue/yellow dimers), in each case representing a set of seven molecules translated along the projection axis **c**.

As explained in §[Sec sec3.7]3.7, the Hyp-1 dimers form a pillar following a left-handed helical line with a pitch of *c*/7 (black line in Figs. 7 ▶ *a* and 7 ▶ *b*). The ANS molecules follow the helical pattern of the protein dimers but can be segregated into three groups. The first group (yellow in Figs. 7 ▶ *a* and 7 ▶ *b*), corresponding to binding sites 1 and 2, are located within the protein cavities and are closely associated with unique protein partners and therefore exactly follow the protein helix. The molecules in the second group correspond to binding sites 7 and 8 (green), where they link Hyp-1 molecules, helping to create the helix of dimers. The ANS molecules in the third group (red) lie outside of the protein helix and at sites 4, 5 and 6 glue the neighbouring helices together. This group also includes the surface-pocket site 3. The red molecules follow a (red) helical line that is similar to that of the Hyp-1 helix but has a larger radius. The ANS molecules viewed along the helical axis are shown in Fig. 7 ▶(*b*). Even though they follow the respective helical lines, they do not create a regular angular pattern around the helix axis.

### 3.6. ANS binding   

Although the ANS ligand was added to the crystallization buffer as a DMSO solution of the acid form (sulfonic acid), there is no doubt that in view of the p*K*_a_ value of −1 the compound is deprotonated to its anionic form (sulfonate) in aqueous solutions and upon interaction with a protein.

#### 3.6.1. Hyp-1–ANS binding assays   

**Figure 8:**
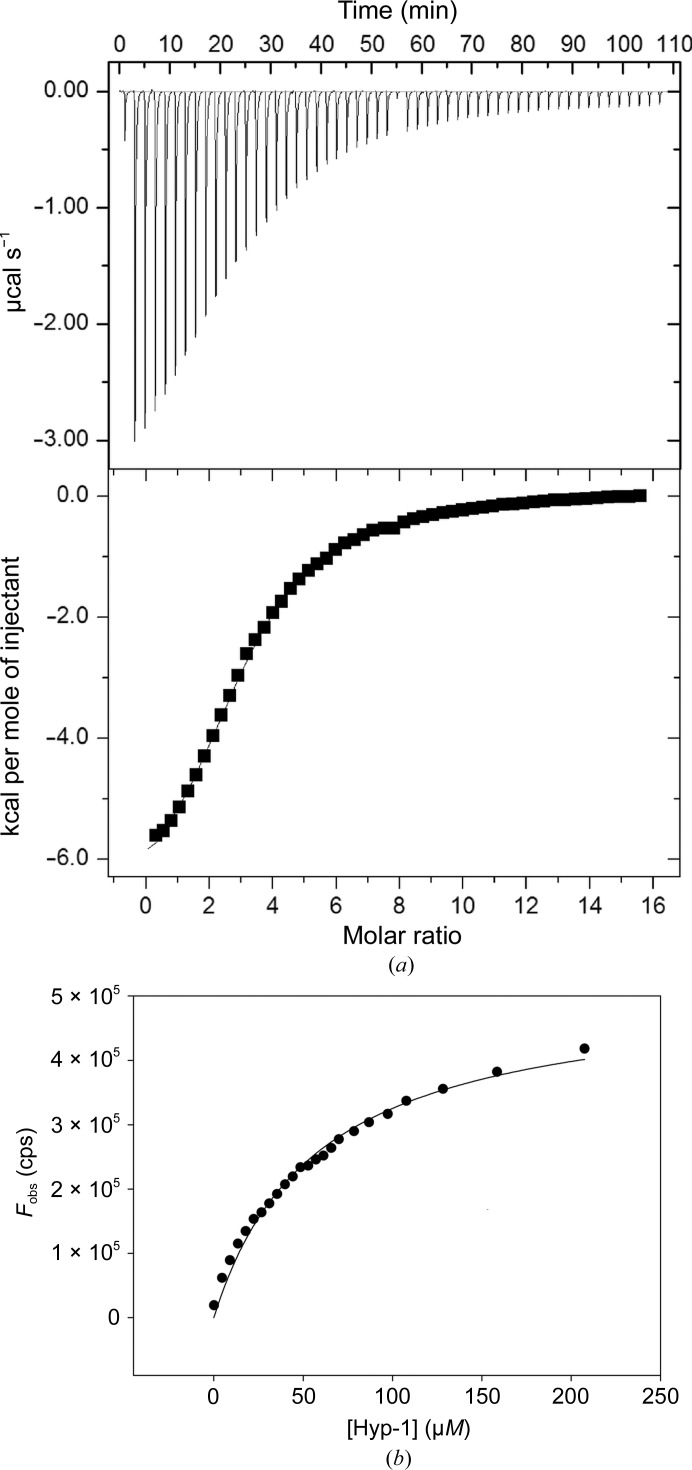
(*a*) ITC titration of Hyp-1 with ANS. The top panel shows raw heat data for 54 consecutive injections of 5 m*M* ANS into the sample cell (200 µl) containing 0.145 m*M* Hyp-1 in 25 m*M* HEPES pH 7.4 at 298 K. The bottom panel shows the binding isotherm created by plotting the heat peak areas against the molar ratio of ANS added to the protein. The line represents the best fit to the model of *N* independent sites. ANS binding is endothermic with 1:3 stoichiometry (*N* = 3.14 ± 0.02) and a *K*_d_ of 108 ± 3 µ*M*. The change in enthalpy Δ*H* is −7213 ± 77 cal mol^−1^ and that in entropy Δ*S* is −6.04 cal mol^−1^ K^−1^. (*b*) ANS binding to Hyp-1 monitored fluorometrically by titration of 1 µ*M* ANS in 25 m*M* HEPES pH 7.4 with Hyp-1. The line represents the best fit to the equation *F* = *F*_max_[protein]/(*K*_d_ + [protein]). The calculated *K*_d_ value is 58 ± 4 µ*M* (*R* ^2^ of fitting 0.9878).

ANS binding by Hyp-1 in solution was tested by both calorimetric and fluorometric assays. The calorimetric titration curve (Fig. 8 ▶ *a*) was fitted using a model of one set of *N* independent binding sites to yield a stoichiometry of *N* = 3 and a dissociation constant *K*_d_ = 108 ± 3 µ*M*. At the end of the ITC titration, when all three binding sites were saturated, the Hyp-1:ANS molar ratio was 1:12. We note that the eightfold molar excess of the ligand during the crystallization experiments resulted in incomplete occupation (2.14 per protein molecule on average) of the three binding sites, although on the other hand as many as 29 interstitial ANS molecules were still available for docking. It is difficult, however, to directly compare the situation within a crystal lattice with the dynamic equilibrium in solution.

In fluorometric titration, the titration system is inverted and we used a fluorescent ligand at a very low and constant concentration together with a variable concentration of the protein. In such a system, where the ligand concentration is much lower than the expected *K*_d_, we do not achieve full saturation of the protein with the ligand. Moreover, if one of the sites has a much higher affinity, the *K*_d_ value determined in such an assay could refer to that particular site only. From the fluorometric titration of ANS with Hyp-1 (Fig. 8 ▶ *b*), a *K*_d_ value of 58 ± 4 µ*M* was determined, which is in reasonable agreement with the global value from the ITC experiment. From the analysis of the crystal structure it could be speculated that ANS binding at site 1 is the strongest, as the protein always uses Arg27 to form an ion pair with the ligand with the same binding geometry, in contrast to sites 2 and 3 where mainly hydrophobic interactions are detected supported by sporadic hydrogen bonds. It is therefore likely that the *K*_d_ value of 58 ± 4 µ*M* most closely characterizes site 1.

#### 3.6.2. Structural description of the ANS sites   

As mentioned above, in addition to the three (internal) ANS docking sites (1, 2 and 3) there are also interstitial sites 4, 5, 6, 7 and 8 occupied by ANS molecules that ‘glue together’ some of the Hyp-1 molecules in the crystal structure. Hereafter, the ANS sites are denoted using the protein chain label (of the nearest protein molecule for interstitial sites) and the site number, *e.g.* *A*1.

#### 3.6.3. Internal Hyp-1 ligand-binding sites   

Binding sites 1 and 2 are internal enclosures or chambers within a general PR-10-type cavity that are sealed off and separated from one another. In fact, a typical PR-10 cavity is not present in the Hyp-1 core because the two chambers are nearly completely isolated and binding sites 1 and 2 have their own separate entrances: E1 and E2, respectively. Entrance E1 is surrounded by loops L3, L5 and L7 and the N-terminal part of helix α3, whereas entrance E2 is gated by the full length of α3 and strand β1. The main partition between sites 1 and 2 is formed by Arg27 from helix α2. Additional residues that form a division between sites 1 and 2 are Ala140 and Phe143 from helix α3, Tyr84 from strand β6 and Tyr101 from strand β7. As a consequence, there is no contact between the ANS molecules at sites 1 and 2. Site 3 is a deep surface-binding pocket formed by a deep invagination of the protein surface between Lys33 and Tyr150.

It is intriguing to note that in the numerous (28) copies of the protein molecule, a given binding site is either fully occupied by an ordered ANS molecule (the most typical situation) or is left completely empty. With just one exception (site *R*3 with 50% occupancy), there are no intermediate situations observed, for example of partial occupancy of a binding site or of a snapshot of an ANS molecule during its transition to its final binding site.

**Figure 9:**
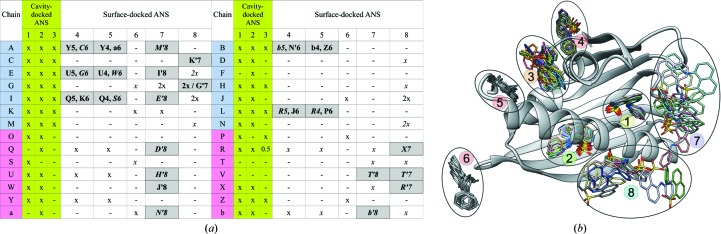
(*a*) Saturation of the Hyp-1 molecules in the two rows, highlighted in blue (I) and pink (II), with ANS ligands. Full occupancy is marked with an x (or 2x if two ligand molecules are found in the general area of a particular binding site), 0.5 denotes a single case (site *R*3) of 0.5 occupancy. The docked sites 1, 2 and 3 are highlighted in green. An entry on a grey background with designation of contact sites in adjacent (one or two) Hyp-1 molecules repeats another entry marked x. Symmetry-related protein molecules are indicated with primes. ANS molecules farther than 3.2 Å from a particular protein chain are marked in italics. (*b*) All of the ANS molecules (sticks) superposed using a common frame of the C^α^ atoms of the nearest protein molecule (shown in ribbon representation). The intramolecular binding sites 1, 2 and 3 are much more constant than the superficial sites, especially 7 and 8. The ANS molecules are colour-coded by the nearest protein molecules in Fig. 6 ▶(*a*).

From the point of view of saturation with the ANS ligand, the two protein rows related in the asymmetric unit by the noncrystallographic 2_1_ screw axis along **c** are not equivalent at all (Fig. 9 ▶ *a*). In row I (dimers *AB*/*CD*/*EF*/*GH*/*IJ*/*KL*/*MN*), the ‘first’ Hyp-1 molecule of each dimer (*A*, *C*, …, *M*) has the internal docking sites 1, 2 and 3 fully saturated with ANS in all cases and the ‘second’ molecules (*B*, *D*, …, *N*) are nearly all fully saturated, with the only vacancies left at *D*3, *F*1, *F*3, *J*3 and *N*3. The situation in row II (*OP*/*QR*/*ST*/*UV*/*WX*/*YZ*/*ab*) is very different. Here, the first Hyp-1 molecules (*O*, *Q*, …, *a*) have many vacancies, with site 3 being empty in all of them (with additional vacancies at sites *Q*1, *S*2 and *a*1). The set of the second molecules (*P*, *R*, …, *b*) of these dimers has nearly the same number of vacancies but with an entirely different pattern, namely with Hyp-1 molecules *T* and *V* having no internal ligands and with additional vacancies at site 3 of *R* (partial), *X* and *b* and at site 2 of *P*.

Considering all of the internal sites of all the Hyp-1 molecules in both rows, it can be summarized that site 1 is empty in five cases, site 2 in four cases and site 3 in 15 cases (15.5 to be exact). Most vacancies (19.5 out of 24.5) are in row II. It appears that this unusual and complicated pattern of docked ANS ligands in the two rows of Hyp-1 molecules repeats itself regularly throughout the crystal lattice because the electron density of the ANS molecules at these sites is very good, clearly indicating well conserved unique orientations and conformations of the ligands.

Table 3 ▶ illustrates the interactions between protein residues and the ANS molecules at sites 1, 2 and 3. The ANS molecule at site 1 is mainly anchored by a salt bridge between the sulfonate anion and the guanidinium group of Arg27 (Fig. 4 ▶ *a*). In two cases, ANS at site 1 is additionally pushed from the outside by hydrophobic contacts with an external ANS molecule at site 7. The main molecular contact at site 2 is based on stacking interactions between the aniline substituent of ANS and the aromatic ring of Tyr144, supported in 11 copies of Hyp-1 by hydrogen bonding to the N^ζ^ atom of Lys8 from strand β1, which also delimits this binding pocket. The ligand molecule at site 3 forms vice-type stacking interactions with Lys33 and Tyr150, which additionally form hydrogen bonds to the ANS molecule in one and eight cases, respectively. As ANS binding to proteins is mainly affected by ionic interactions with positively charged residues (Matulis & Lovrien, 1998 ▶), one can speculate that in Hyp-1 binding site 1 the dominating interaction is with the positive charge of Arg27. At site 2, this role could be played by Lys8, which in about half of the cases is in hydrogen-bonding contact with the ligand. At site 3, Lys22 is the nearest cationic centre but it forms a hydrogen-bond contact with ANS in only one case.

#### 3.6.4. Interstitial ligands   

The 29 interstitial ANS molecules occupy the five superficial sites (4–8) on the surface of the protein molecules much more sparsely and there does not seem to be a discernible pattern of occupancy. The sparsity of the superficial sites is similar around both rows. There is no Hyp-1 molecule that has all of the associated superficial sites occupied. Likewise, none of the superficial sites is occupied in all copies of the protein. Moreover, while the internal sites are always occupied in exactly the same manner, leading to very good superposition of the ligand molecules, particularly at sites 1 and 2 (Fig. 9 ▶ *b*), the superficial positions show a higher degree of positional and conformational variability, which at sites 7 and 8 is manifested by a range of locations.

The interstitial ANS molecules in sites 4, 5 and 6 are surrounded by three neighbouring protein chains and are stabilized mainly by hydrogen bonds to the peptide group of Gly47 (in loop L4). This interaction is supported in several cases by contacts (<3.2 Å) with single atoms from loops L6 and L8. The ANS molecules at sites 7 and 8, where they glue two adjacent protein molecules, interact with protein residues from loops L3 and L5 as well as from helix α3. Residue Lys138, which in most cases forms a salt bridge to the sulfonate group, seems to play a crucial role in these interactions.

Fig. 9 ▶(*b*) shows all 89 ANS molecules superposed using a common C^α^ framework of the nearest Hyp-1 molecule. It indicates that the position of the ligand molecule is most stable at sites 1, 2, 4, 5 and 6. At site 3 the ANS molecule appears to be rotating between the jaws of the vice. Sites 7 and 8 are characterized by a large scatter. However, the pattern is not random but is located alongside helix α3 (8) and the E1 entrance (7) of a neighbouring protein molecule.

#### 3.6.5. ANS conformation   

The geometry of the ANS molecules^1^ was analyzed using the three rotatable torsion angles τ_1_ (C2—C1—S—O; the orientation of the sulfonate group), τ_2_ (C7—C8—N—C11; the orientation of the aniline substituent) and τ_3_ (C8—N—C11—C; the rotation of the phenyl ring of the aniline substituent). Table 4 ▶ illustrates that the conformations at the different binding sites are quite distinct, with the exception of the τ_1_ angle, which owing to the threefold symmetry of the substituent is generally close to 0°. The ANS molecules at sites 1 and 2 have well conserved but different conformations. In particular, the aniline substituent at site 1 deviates from the naphthalene plane in a very significant way. The rotational variability of the phenyl substituent is quite large, especially at sites 3 and 7/8, as illustrated by the elevated values of the standard deviations in Table 4 ▶. This agrees with the observation that while the vast majority of the ANS molecules have perfect definition in the electron density, in seven cases (five of which are at sites 7 and 8) the electron density of the aniline substituent is blurred.

Although the torsion angles τ_1_ and τ_3_ of the ANS molecules are similar to those in the ANAPHS structure from the CSD, the τ_2_ angle deviates quite significantly (up to 92°).

The 1,8-substituted naphthalene ring in the small-molecule ANAPHS structure (Cody & Hazel, 1977 ▶) that served as the source of the ANS restraints is significantly distorted, with the substituents showing particularly large deviations from the naphthalene system. The weight of planarity restraints (σ_flat_ = 0.02 Å) applied in *REFMAC* evidently over-restrained the planarity against the experimental evidence, visible for example as a >10σ deviation from planarity of the N atom in 33 ANS molecules. An additional round of refinement with σ_flat_ = 0.2 Å was able to rectify this and created ANS models with similar deviations from idealized geometry as in ANAPHS. The issue of ANS deformations will be analyzed in depth elsewhere.

### 3.7. Pseudosymmetric aspects of the crystal structure   

The crystal structure of Hyp-1–ANS is highly pseudosymmetric in two aspects: firstly because of the way the protein molecules are arranged in infinite columns along the longest cell dimension and secondly because of the way these columns pack in the unit cell.

Fig. 6 ▶(*a*) presents the 28 Hyp-1 molecules grouped into columns built from the pseudo-twofold-symmetric dimers *AB*, *CD*, …, *ab* that are arranged in a zigzag fashion. One half of this column (row I) is formed by (seven) dimers *AB*, …, *MN* separated by a shift of ∼1/7 of the cell length. The second half of the column (row II) is formed by a similar series of dimers *OP*, …, *ab* and can be generated from the first row by a rotation of ∼180° and a translation of ∼1/2 along the **c** axis, which is equivalent to a translation of ∼1/2 of the interdimer distance. The column composed of these two rows of dimers (red/green and blue/yellow in Fig. 7 ▶ *c*) is therefore formed according to a ‘2_1/7_’ screw axis, with a rotation of ∼180° and a translation of ∼1/14 along the unit-cell **c** axis. In addition, there are two sets of pseudo-twofold axes perpendicular to the column axis. The Hyp-1 dimers are generated by one set and there are 14 such dyads in the unit cell. The dimers across the zigzag pattern of the column are related by the axes from the second set and there are also 14 such dyads; they are perpendicular to the first set and are located halfway between them. The approximate symmetry of the column may be described by the symbol 222_1/7_. The distances between the successive Hyp-1 molecules are similar but not equal, and the location of the ANS ligands is also variable. Perfect repetition along the column is achieved only after seven translations.

If the 28 Hyp-1 molecules are collapsed to a sevenfold smaller unit cell, *i.e.* if all dimers are shifted by the appropriate fraction of the *c* cell length (1/7, 2/7 *etc.*) and overlapped on the *AB* and *ab* dimers, the r.m.s. distance of all 4452 C^α^ atoms from their mean position in each group of seven molecules is 1.18 Å. The symmetry of such an assembly is approximately 222_1_. If, in addition, all of the molecules are transformed according to that symmetry, the 28 molecules superpose onto one target with an r.m.s.d. of 1.23 Å. The latter value illustrates the difference between the positions of all of the C^α^ atoms in the real (pseudosymmetric) and idealized (222_1/7_ symmetric) column.

In the *C*2 unit cell there are four columns of Hyp-1 molecules as described above. Owing to their pseudosymmetry, their packing is also pseudosymmetric, as illustrated in Fig. 7 ▶(*c*). After an appropriate shift along the monoclinic *y* axis, the four columns are positioned exactly in each of the four quarters of the unit cell, and at a cursory glance their packing seems tetragonal. Indeed, the *C*-centring moves the ¼, ¼ column to ¾, ¾, and these two columns are related by a 2_1/7_ axis, which also includes a 2_1_ operation (as its sevenfold repetition). Similarly, the monoclinic twofold axis transforms the ¼, ¼ column to ¾, ¼ and the monoclinic 2_1_ axis transforms it to ¼, ¾. The columns in the latter two pairs are also related by an approximate 4_−1/7_ screw axis involving a clockwise 90° rotation and a negative shift by 1/28 of the *c* axis. This left-handed 4_−1/7_ screw axis includes a right-handed 4_1_ screw axis (as its sevenfold repetition), a 2_1/7_ screw axis (twofold repetition) and a 2_1_ screw axis (14-fold repetition). These relations are analogous to the case of the left-handed 6_4_ screw axis, which contains the right-handed 3_1_ and neutral 2_1_ screw axes (Dauter & Jaskolski, 2010 ▶).

Taking into account the presence of the (perpendicular) twofold axes, the arrangement of the Hyp-1 molecules in the four columns approximately corresponds to the *P*4_1_22 and ‘*P*4_−1/7_22’ space groups. The primitive tetragonal unit cell has one-half of the *C*2 cell volume and is rotated by 45° around **c**. In addition, to conform to the location of the twofold axes in the original *C*2 symmetry, the origin of the tetragonal cell is shifted along the fourfold axis by −1/8. The handedness of the pseudo-tetragonal axis results from the particular shift of the Hyp-1 columns with their local dyads with respect to the crystallographic twofold axes. If the columns were shifted from the current position by an odd multiple of 1/28 of the cell length (1/28, 3/28, …, 7/28 = 1/4, …), the pseudo-tetragonal space group would be *P*4_3_22 or ‘*P*4_1/7_22’. If all Hyp-1 molecules in the four columns are superposed onto one target according to the idealized *P*4_−1/7_22 symmetry, the r.m.s.d. value for all C^α^ atoms is 1.71 Å.

A less intuitive view of the crystal packing, but one that is more amenable to analysis, is obtained by considering four rows of Hyp-1 dimers extending along **c** as a ‘pillar’. In this view, the protein dimers in such a pillar follow a left-handed helical line in the order *ba*–*NM*–*AB*–*OP*–*ZY*–*LK*–*CD*–*QR*–*XW*–*JI*–*EF*–*ST*–*VU*–*HG* within 0 ≤ *z* < 0.5 and then continue smoothly in the unit cell (0.5 ≤ *z* <1) in the order *GH*–*UV*–*­TS*–*FE*–*IJ*–*WX*–*RQ*–*DC*–*KL*–*YZ*–*PO*–*BA*–*MN*–*ab*. The pillar (around the grey 4_1_ axis in its centre) viewed along its axis can be seen in Fig. 7 ▶(*c*). The helical line of the protein packing can be traced through the centres (mean coordinate) of the main-chain atoms of each dimer in the pillar. Each dimer is rotated 90° counterclockwise around the helical axis and translated by 1/28 of the **c** parameter with respect to the previous point. This helical line (black in Figs. 7 ▶ *a* and 7 ▶ *b*) has a pitch of *c*/7, *i.e.* it is commensurate with the *c* axis (has seven periods in one **c** repeat) and runs as a smooth wave over the Hyp-1 dimers from one unit cell to the next.

The square shape of the unit-cell base and the highly pseudo-tetragonal character of the arrangement of Hyp-1 molecules are conducive to ‘erroneous’ packing of the Hyp-1 columns in different unit cells without significant distortions or dislocations in the crystal. This explains the occurrence of tetartohedral twinning, in which individual domains of the crystal are related by fourfold rotation around the long cell axis.

The pseudosymmetry of the packing of the Hyp-1 molecules strongly influences the intensity of diffraction. This is visible not only in the sevenfold modulation illustrated in Fig. 2 ▶(*c*), but also in the values of the structure factors related by the pseudo-tetragonal symmetry. Since the crystal of Hyp-1–ANS was perfectly tetartohedrally twinned, the measured intensities *I*_obs_ conform to 422 symmetry with an *R*_merge_ of 7.5%. To eliminate the effect of twinning, *R*_merge_ was also calculated using *I*_calc_ values obtained after refinement and this value was 26%, significantly less than the value of about 50% usually obtained for merging data in the wrong symmetry.

Normally, the *R* factors resulting from structure refinement against merohedrally twinned data are lower than expected for nontwinned crystals; whereas a completely wrong model with randomly positioned atoms gives an *R* factor of 58% for untwinned crystals (Wilson, 1950 ▶), for hemihedrally twinned crystals this value is 41% (Murshudov, 2011 ▶). From this perspective, the *R* and *R*_free_ values of 22.3 and 27.8%, respectively, which would be quite normal for a ‘healthy’ structure at 2.43 Å resolution, might seem somewhat high for a highly twinned crystal. However, the analysis of Murshudov (2011 ▶) corresponds to twinned structures with random distributions of atoms in the unit cell. Contrary to this assumption, the structure of Hyp-1–ANS is highly pseudosymmetric, with atoms distributed in a nearly tetragonal fashion, despite the true monoclinic *C*2 space group. As a result of this pseudo-tetragonal arrangement, the reflections related by 422 point-group symmetry operations have related intensities, as illustrated by the above *R*_merge_ of 26% calculated using *I*_calc_, *i.e.* corresponding to pseudosymmetric but untwinned data. The *F*_calc_ statistics are opposite to those expected for twinning, with larger than normal fractions of very weak and very strong data, as is characteristic for tNCS. The twin laws (which also correspond to 422 symmetry) therefore mix reflections that are similar by pseudosymmetry, rather than mixing unrelated contributions from different twin domains. This explains why various twinning criteria, including the *L*-test, did not clearly indicate the presence of a very high degree of twinning in the experimental set of intensities. For this reason, for twinned but highly pseudosymmetric crystals the refinement *R* factor will not be expected to be much lower than for ordinary structures, and in this context the value of ∼22% for such a huge structure as Hyp-1–ANS should be considered to be quite normal. The correctness of the refined model is further confirmed by the distributions of the scale (close to ∼1) and *R* factors (inversely related to average reflection layer intensity) in seven *n* = mod(*l*, 7) groups calculated in different resolution ranges (Supplementary Table S1). Also, the CC_work_ and CC_free_ coefficients, when compared with CC*, show the expected behaviour, with slight fluctuation in pace with the overall intensity of the subsets considered (Supplementary Table S2).

### 3.8. Comparison with other PR-10 proteins   

#### 3.8.1. Superpositions of the present Hyp-1 models   

Structural comparisons of the 28 Hyp-1 models from the present structure show that they are all very similar. In particular, there are no meaningful differences between the C^α^ traces of the Hyp-1 molecules that are fully occupied by ANS and those without any ligand. For example, the C^α^ r.m.s.d. for chains *K* (three ANS ligands) and *T* (no ligands) is 0.41 Å, *i.e.* it is very similar to the value of 0.46 Å for the *A*/*K* pair with both chains fully occupied by ANS. This illustrates that there is no conformational adaptation of the Hyp-1 framework upon ligand binding, at least for ligands such as ANS.

#### 3.8.2. Comparison with the unliganded structure of Hyp-1   

The present models of Hyp-1 are also very similar to the previously reported ligand-free form (PDB entry 3ie5; Michalska *et al.*, 2010 ▶), with C^α^ r.m.s.d. values of ∼0.6 Å. In a structural superposition, one notes that the L5 and α2 elements of chain *A* of the PDB entry 3ie5 are tilted toward the cavity when compared with chain *B* from the same structure or with, for example, chain *K* of the present structure, but in general, in agreement with the above conclusion, there are no clear manifestations of structural adaptability upon ANS binding. It should be noted, however, that the formally ligand-free structure with the PDB code 3ie5 in fact has PEG molecules in the binding cavity. Interestingly, the PEG molecules occupy similar sites as ANS ligands 1, 2 and 3 in the present structure, suggesting conservation of these Hyp-1 binding sites. Also, the residues responsible for ligand interactions (<3.2 Å) in the PDB entry 3ie5, Lys8 and Lys33 of chain *A* and Arg27 and Gln35 of chain *B*, are the same as those involved in ANS binding (Table 3 ▶).

#### 3.8.3. Comparison of Hyp-1 with other PR-10 models   

The structure of the Hyp-1–ANS complex reveals an interesting location of ligand-binding sites that is not found in other PR-10 proteins. The structures of PR-10 complexes reported to date have either a huge hydrophobic cavity which spans the entire space between the E1 and E2 entrances or have a small cavity with only one entrance, E1. The former group, represented by proteins such as the birch allergen Bet v 1 (*e.g.* PDB entry 4a80; Kofler *et al.*, 2012 ▶), PR-10 isoforms from yellow lupin (PDB entries 1icx, 1ifv, 1xdf and 2qim; Biesiadka *et al.*, 2002; Pasternak *et al.*, 2005 ▶; Fernandes *et al.*, 2008 ▶) or SPE16 from jicama (PDB entry 1txc; F. Wu, Z. Wei, Z. Zhou & W. Gong, unpublished work), can accommodate more than two ligand molecules with many hydrophobic contacts, whereas the latter group, represented by phytohormone-binding proteins (PhBP) from *Vigna radiata* (PDB entry 2flh; Pasternak *et al.*, 2006 ▶) and *M. truncatula* (PDB entry 4q0k; Ruszkowski *et al.*, 2014 ▶) and by *M. truncatula* nodulin 13 (PDB entry 4jhg; Ruszkowski *et al.*, 2013 ▶), usually bind only one ligand molecule, typically *via* hydrogen bonding. The two internal binding sites of Hyp-1, each with a separate entrance, are a novelty that is reported for the PR-10 proteins for the first time. Also, the deep surface-invagination binding pocket 3 is a novel feature. The C^α^ r.m.s.d. values between chain *K* of the present structure and PDB entries belonging to the two PR-10 groups mentioned above are rather high (typically 1.8 Å or more) and are similar for both groups (Table 5 ▶).

#### 3.8.4. ANS and other PR-10 ligands   

A growing number of crystal structures of small-molecule complexes of PR-10 proteins underscore their ability to bind various physiolo­gically important molecules such as cytokinins, gibberellins, abscisic acid, steroids or flavonoids. These accumulating observations need to be verified in solution to eliminate the possibility of crystallographic artifacts and to characterize the complexes kinetically. ANS as a fluorescence probe, with its aromatic ring and small size, is an excellent mimic of the above natural ligands for such studies.

#### 3.8.5. Comparison of ANS binding in PR-10 complexes   

**Figure 10:**
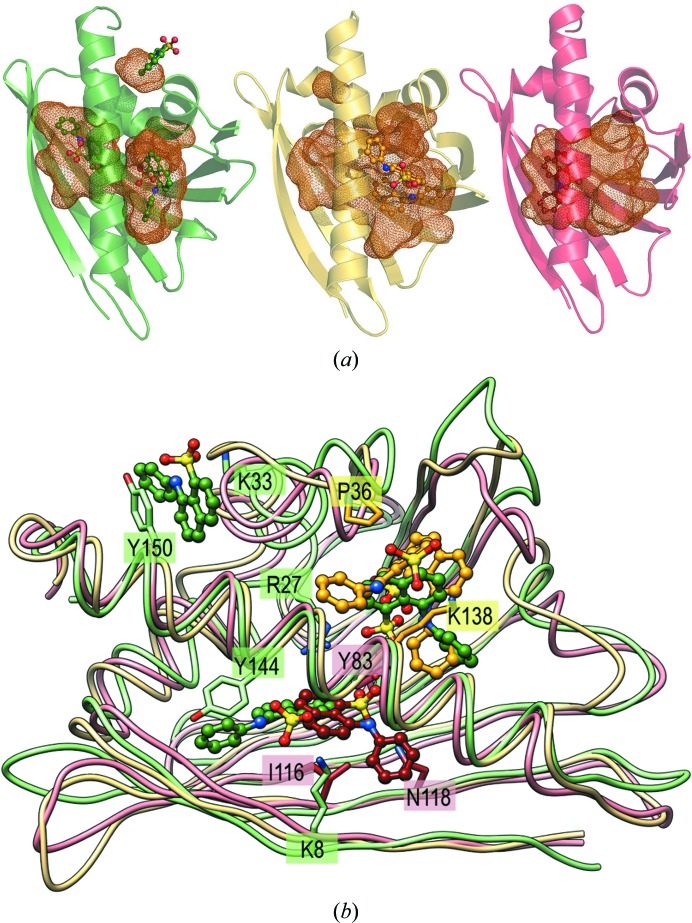
(*a*) ANS molecules within the differently shaped cavities (shown in van der Waals mesh representation) of the present Hyp-1 chain L (green), 1txc (yellow) and 4a80 (red). All the available sites are probed by the ligand only in the Hyp-1 complex. (*b*) Structural alignment of these protein chains (colour coded as in *a*), with highlighting of residues involved in ANS contacts (<3.2 Å).

To date, two other PR-10 proteins have been crystallized in complex with ANS, namely isoforms a (PDB entry 4a80) and j (PDB entry 4a8v) of Bet v 1 from birch pollen (Kofler *et al.*, 2012 ▶), with one ANS molecule in the same position near the E2 entrance to the cavity (corresponding roughly to the present site 2), and SPE16 from jicama with two ANS molecules near the E1 entrance (corresponding roughly to the present site 1), which was deposited in the PDB (as entry 1txc) without publication. Superposition of those two structures with Hyp-1–ANS (represented by chain *L*) shows that all three potential binding sites are only occupied in Hyp-1. Moreover, in the case of Bet v 1, additional structural data revealed that natural ligands are bound in a binding site that is not occupied by ANS (Kofler *et al.*, 2012 ▶). Mapping of the binding cavities with van der Waals surfaces (Figs. 10 ▶ *a* and 4 ▶ *b*) shows that only in Hyp-1 are they structurally well defined and distinct, which is of advantage in the interpretation of ADA results, as no direct interactions can be expected between ligands in different binding sites.

Structural alignment of Hyp-1 (chain *L*) with PDB entries 1txc and 4a80 (Fig. 10 ▶ *b*), with highlighting of the residues involved in ANS contacts (<3.2 Å), shows that binding site 1 of Hyp-1 has no common residues with PDB entry 1txc. Intriguingly, the conserved residues Lys33 and Tyr150 that form the vice of Hyp-1 site 3 make no ligand interactions in the two other structures.

## 4. Conclusions   

A co-crystallization experiment produced tetartohedrally twinned, highly pseudosymmetric Hyp-1–ANS crystals with a modulated superstructure. The modulation is manifested by intensity fluctuations in reciprocal space, with crests at *l* = 7*n* and *l* = 7*n* ± 3. In direct space, a group of four Hyp-1 molecules (with pseudotetragonal packing) is sevenfold repeated along **c**. Since the modulation appears to be commensurate, the structure could be successfully refined and interpreted in an expanded (sevenfold along **c**) supercell. Because of the severe twinning, the structure was solved by MR using a tNCS-corrected ML algorithm in triclinic symmetry searching for 56 protein molecules, and the correct space group (*C*2) was figured out (in reciprocal space) by analyzing the *P*1 solution. The final model is of high quality and reveales an unusual mode of ligand binding consisting of two internal sites and a deep pocket on the surface of the Hyp-1 molecule. The 1:3 complex was characterized in solution by fluorometric and calorimetric measurements. In addition to 60 protein-docked ligands, there are 29 interstitial ANS molecules distributed in a pattern that violates the arrangement of the protein molecules and is likely to be the generator of structural modulation. In particular, the tNCS-related Hyp-1 molecules are found closer together whenever there is an ANS molecule linking them. Twinning detection is very difficult in the presence of tNCS and is further complicated by additional rotational pseudosymmetry (Lebedev *et al.*, 2006 ▶; Zwart *et al.*, 2008 ▶). The strength of twinning tests could be analyzed without ambiguity, as the twinning in this case is noncontroversial because of the prohibited symmetry displayed by the diffraction pattern.

## Supplementary Material

PDB reference: Hyp-1, a St John’s wort PR-10 protein in complex with the fluorescent probe 8-anilino-1-naphthalene sulfonate, 4n3e

Supplementary Material.. DOI: 10.1107/S1399004715001388/tz5069sup1.pdf

## Figures and Tables

**Table 1:** Refinement statistics Values in parentheses are for the last resolution shell.

Space group	*C*2
Unit-cell parameters (, )	*a* = *b* = 146.21, *c* = 298.35, = 90.07
Data-collection temperature (K)	100
Resolution ()	302.43 (2.472.43)
Reflections measured	496579
Unique reflections	170447†/61810‡
Completeness (%)	99.8 (99.2)§
Multiplicity	8.0 (7.1)§
Refinement program	*REFMAC*5
Unique reflections (work + test)	232268§
Test reflections	3077
Molecules in asymmetric unit	
Protein	28
ANS	89
No. of atoms	
Protein	35224
Ligand	1899
Water	35
Metal	15
*B* (^2^)	
Protein	47.3
Ligand	39.9
Water	27.6
Metal	44.0
*R*_work_/*R*_free_ (%)	22.3/27.8
R.m.s.d. from ideal geometry	
Bond lengths ()	0.015
Bond angles ()	2.18
Ramachandran statistics¶ (%)	
Favoured	91.80
Allowed	7.04
Outliers	1.16
PDB code	4n3e

† Scaled in *C*2 symmetry.

‡ Scaled in *P*422 symmetry.

§ After expansion from *P*422 symmetry.

¶ Assessed with *MolProbity* (Chen *et al.*, 2010 ▶).

**(a):** Analysis of *P*1 model symmetry based on calculated structure factors.

Symmetry element	Symmetry operator	Correlation coefficient	*R*_meas_ †
Twofold *h*	*h*, *k*, *l*	0.93	0.219
Twofold *k*	*h*, *k*, *l*	0.95	0.171
Twofold *l*	*h*, *k*, *l*	0.92	0.241
Twofold diagonal	*k*, *h*, *l*	0.99	0.089
Twofold diagonal	*k*, *h*, *l*	0.92	0.236
Fourfold *l*	*k*, *h*, *l*; *k*, *h*, *l*; *h*, *k*, *l*	0.94	0.228

**(b):** Analysis of *C*2 model symmetry based on calculated structure factors in the *P*422 setting.

		Correlation coefficient		*R*_meas_	
Symmetry element	Symmetry operator	Rigid body	Final	Rigid body	Final
Twofold *h*	*h*, *k*, *l*	0.95	0.67	0.187	0.363
Twofold *k*	*h*, *k*, *l*	0.94	0.70	0.196	0.350
Twofold *l*	*h*, *k*, *l*	0.94	0.75	0.127	0.177
Twofold diagonal	*k*, *h*, *l*	0.92	0.76	0.235	0.348
Fourfold *l*	*k*, *h*, *l*; *k*, *h*, *l*; *h*, *k*, *l*	0.95	0.65	0.187	0.424

† *R*_meas_ = 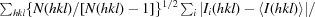 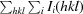 (Diederichs Karplus, 1997 ▶).

**Table 3:** ProteinANS interactions (3.2) in all 28 copies of Hyp-1 at the internal sites 1, 2 and 3 with the frequency in parentheses Residues highlighted in bold form hydrogen bonds to the sulfonate group of ANS (note that Arg27 at site 1 always interacts with ANS *via* hydrogen bonding, while other residues in bold show a variable pattern of hydrogen-bonding/hydrophobic/no interactions at the remaining binding sites).

Binding site (No. of chains occupied by ANS in this binding site)	Residues involved in contact to ANS (No. of chains with this interaction)
1 (23)	**Arg27** (22), Gln35 (5), Leu31 (2), Val91 (1), Glu132 (1), Gly136 (1), Lys139 (1), ANS at site 7 (2)
2 (24)	Tyr144 (23), **Lys8** (12), Leu19 (6), Ile116 (6), **Glu10** (5), Tyr141 (3), Leu23 (2), Arg27 (1), Tyr84 (1)
3 (13)	**Tyr150** (11), **Lys33** (9), Val30 (3), Val147 (3), Phe158 (3), Val157 (2), Leu151 (1)

**Table 4:** Statistics of the conformations (torsion angles _1_, _2_ and _3_) of the ANS molecules at different Hyp-1 binding sites compared with the CSD structure ANAPHS (Cody Hazel, 1977 ▶) For each angle at the designated sites, the mean value and its standard deviation are given. Sites 4, 5 and 6 are treated jointly as they correspond to essentially the same position of the ligand at which it glues together three neighbouring Hyp-1 chains. Likewise, sites 7 and 8 are found between two Hyp-1 chains. The statistical analysis takes into account the discontinuity (+180/180) in torsion-angle definition. The atom numbering of the ANS molecule (Fig. 4 ▶ *c*) follows the recommendation of IUPAC, as explained by Jaskolski (2013 ▶), regardless of the system adopted by the PDB.

	Torsion angle ()					
Site	1	2	3	4/5/6	7/8	ANAPHS
C2C1SO† (_1_)	3 (2)	1 (2)	2 (2)	5 (2)	1 (2)	1
C7C8NC11 (_2_)	38 (2)	12 (1)	2 (6)	21 (5)	11 (6)	54
C8NC11C‡ (_3_)	11 (3)	24 (5)	3 (11)	31 (4)	1 (12)	7

† The sulfonyl O atom was selected to minimize |_1_|.

‡ The aniline C12 atom was selected to minimize |_3_|.

**Table 5:** C r.m.s.d. values between chain *K* of the present structure and PR-10 models (identified by their PDB codes) with a large hydrophobic void (I) or a small cavity with one entrance (II)

	Protein	R.m.s.d. ()
I	4a80 (Bet v 1)	1.57
	1icx (LlPR-10.1A)	1.83
	1ifv (LlPR-10.1B)	1.94
	1xdf (LlPR-10.2A)	2.08
	2qim (LlPR-10.2B)	1.74
	1txc (SPE16)	1.56
II	2flh (VrPhBP)	2.36
	4q0k (MtPhBP)	1.48
	4jhg (MtN13)	1.93

## Footnotes

1 The numbering scheme of the ANS molecule (Fig. 4 ▶ *c*) follows the recommendation of IUPAC, as explained by Jaskolski (2013 ▶), regardless of the system adopted by the PDB.
