# Supplementary material for: ANS complex of St John’s wort PR-10 protein with 28 copies in the asymmetric unit: a fiendish combination of pseudosymmetry with tetartohedral twinning
Source: Acta Crystallogr D Biol Crystallogr. 2015 Mar 26;71(Pt 4):829–43. doi: 10.1107/S1399004715001388 (PMC4388264; doi:10.1107/S1399004715001388)
Supplement: Supplementary file 1 [file d-71-00829-sup1.pdf]

BIOLOGICAL
CRYSTALLOGRAPHY

Volume 71 (2015)

Supporting information for article:

ANS complex of St John's wort PR-10 protein with 28 copies in the asymmetric unit: a fiendish combination of pseudosymmetry with tetartohedral twinning

Joanna Sliwiak, Zbigniew Dauter, Marcin Kowiel, Airlie J. McCoy, Randy J. Read and Mariusz Jaskolski

Table S1 Scale and *R* factors in seven L=mod(*l*,7) groups, calculated in different resolution ranges (in Å) for FC_ALL_LS structure factors from *REFMAC* output.

scales on FP*FP/FC_ALL_LS*FC_ALL_LS

Resolution	5.250	4.170	3.640	3.310	3.070	2.890	2.740	2.620	2.520	2.430	All
0	0.951	0.996	1.026	1.069	1.009	0.906	0.840	0.800	0.790	0.784	0.978
1	0.983	0.933	1.000	1.020	1.015	0.835	0.810	0.782	0.765	0.796	0.953
2	0.630	1.078	1.246	1.373	1.361	1.117	1.032	0.975	0.965	0.885	1.095
3	1.139	0.974	1.052	1.092	1.069	0.925	0.878	0.846	0.820	0.804	1.035
4	1.062	0.944	1.050	1.029	1.003	0.914	0.825	0.792	0.800	0.789	0.993
5	0.553	1.079	1.292	1.306	1.278	1.051	0.949	0.892	0.882	0.869	1.079
6	0.999	1.054	1.095	1.124	1.106	0.943	0.870	0.876	0.844	0.846	1.045
All	0.996	0.977	1.054	1.077	1.049	0.922	0.856	0.824	0.815	0.809	1.061

scales on FP/FC_ALL_LS

Resolution	5.250	4.170	3.640	3.310	3.070	2.890	2.740	2.620	2.520	2.430	All
0	1.000	1.001	1.005	1.018	1.003	0.949	0.908	0.894	0.897	0.904	0.985
1	0.969	0.969	1.000	1.019	1.024	0.921	0.907	0.904	0.902	0.918	0.968
2	0.785	1.054	1.141	1.208	1.244	1.086	1.059	1.046	1.054	0.984	1.066
3	1.065	0.988	1.029	1.042	1.043	0.961	0.938	0.931	0.915	0.919	1.007
4	1.020	0.978	1.020	1.008	0.999	0.943	0.904	0.891	0.891	0.905	0.982
5	0.739	1.052	1.166	1.179	1.188	1.052	1.011	1.001	1.001	0.970	1.046

6	0.976	1.029	1.064	1.080	1.093	0.989	0.956	0.971	0.963	0.953	1.022
All	0.987	0.998	1.038	1.052	1.054	0.970	0.940	0.933	0.932	0.930	1.024

R factor on FP/FC_ALL_LS

Resolution	5.250	4.170	3.640	3.310	3.070	2.890	2.740	2.620	2.520	2.430	All
0	0.2733	0.1770	0.1954	0.2162	0.2362	0.2137	0.2131	0.1913	0.1741	0.0740	0.2194
1	0.3318	0.2293	0.2406	0.2542	0.2727	0.2145	0.2130	0.2015	0.1799	0.0815	0.2445
2	0.4529	0.3105	0.3064	0.3170	0.3538	0.2295	0.2226	0.2230	0.2270	0.1053	0.3120
3	0.2145	0.1781	0.1927	0.2092	0.2386	0.2015	0.1957	0.1898	0.1763	0.0798	0.2018
4	0.2148	0.1639	0.1740	0.1973	0.2371	0.2070	0.1993	0.1824	0.1752	0.0778	0.1932
5	0.4810	0.3091	0.3075	0.3226	0.3430	0.2311	0.2146	0.2171	0.2055	0.1013	0.3102
6	0.3343	0.2269	0.2522	0.2592	0.2975	0.2243	0.2223	0.2056	0.1895	0.0918	0.2509
All	0.2869	0.2014	0.2174	0.2376	0.2691	0.2167	0.2118	0.2002	0.1881	0.0871	0.2304

Table S2 Tables of $CC_{1/2}$, CC*, CC_{work} and CC_{free} (on intensities) calculated in seven L=mod(l,7) groups and different resolution (Å) ranges.

CC1/2

Resolution	5.250	4.170	3.640	3.310	3.070	2.890	2.740	2.620	2.520	2.430	All
0	0.9944	0.9980	0.9982	0.9968	0.9956	0.9938	0.9839	0.9696	0.9428	0.8673	0.9967
1	0.9988	0.9983	0.9966	0.9921	0.9895	0.9760	0.9452	0.8904	0.8423	0.6747	0.9967
2	0.9967	0.9954	0.9902	0.9857	0.9701	0.9426	0.8896	0.7834	0.6627	0.4506	0.9862
3	0.9991	0.9972	0.9978	0.9970	0.9958	0.9926	0.9838	0.9669	0.9539	0.8195	0.9984
4	0.9988	0.9985	0.9980	0.9968	0.9955	0.9943	0.9872	0.9774	0.9431	0.8781	0.9987
5	0.9890	0.9901	0.9903	0.9841	0.9744	0.9443	0.8732	0.7275	0.6567	0.4017	0.9845
6	0.9975	0.9957	0.9972	0.9936	0.9857	0.9777	0.9370	0.8737	0.7679	0.6463	0.9959
All	0.9965	0.9985	0.9983	0.9971	0.9955	0.9918	0.9786	0.9558	0.9166	0.7907	0.9994

CC*

Resolution	5.250	4.170	3.640	3.310	3.070	2.890	2.740	2.620	2.520	2.430	All
0	0.9986	0.9995	0.9995	0.9992	0.9989	0.9984	0.9959	0.9923	0.9852	0.9638	0.9992
1	0.9997	0.9996	0.9991	0.9980	0.9974	0.9939	0.9858	0.9706	0.9563	0.8977	0.9992
2	0.9992	0.9989	0.9975	0.9964	0.9924	0.9851	0.9704	0.9373	0.8928	0.7882	0.9965
3	0.9998	0.9993	0.9994	0.9993	0.9990	0.9981	0.9959	0.9915	0.9881	0.9491	0.9996
4	0.9997	0.9996	0.9995	0.9992	0.9989	0.9986	0.9968	0.9943	0.9852	0.9670	0.9997
5	0.9972	0.9975	0.9976	0.9960	0.9935	0.9856	0.9656	0.9177	0.8904	0.7571	0.9961
6	0.9994	0.9989	0.9993	0.9984	0.9964	0.9943	0.9836	0.9657	0.9320	0.8861	0.9990
All	0.9991	0.9996	0.9996	0.9993	0.9989	0.9979	0.9946	0.9886	0.9780	0.9397	0.9998

FP/FC_ALL_LS on Intensities

CCwork

Resolution	5.250	4.170	3.640	3.310	3.070	2.890	2.740	2.620	2.520	2.430	All
0	0.611	0.895	0.907	0.916	0.880	0.862	0.857	0.896	0.906	0.987	0.814
1	0.792	0.899	0.880	0.862	0.832	0.830	0.828	0.867	0.906	0.985	0.896
2	0.649	0.807	0.830	0.811	0.764	0.854	0.856	0.873	0.876	0.976	0.822
3	0.885	0.929	0.922	0.917	0.879	0.866	0.876	0.894	0.916	0.985	0.934
4	0.900	0.899	0.938	0.920	0.875	0.873	0.872	0.918	0.930	0.991	0.933
5	0.481	0.817	0.829	0.791	0.772	0.844	0.826	0.862	0.891	0.974	0.821
6	0.829	0.895	0.879	0.871	0.806	0.855	0.833	0.867	0.905	0.980	0.905
All	0.774	0.925	0.938	0.927	0.893	0.885	0.879	0.912	0.921	0.988	0.875

CCfree

Resolution	5.250	4.170	3.640	3.310	3.070	2.890	2.740	2.620	2.520	2.430	All
0	-	0.749	-	-	-	-	-	-	0.779	-	0.819
1	-	0.779	-	-	-	-	-	-	0.733	-	0.853
2	-	0.693	-	-	-	-	-	-	0.694	-	0.783
3	-	0.889	-	-	-	-	-	-	0.704	-	0.926
4	-	0.888	-	-	-	-	-	-	0.782	-	0.934
5	-	0.674	-	-	-	-	-	-	0.733	-	0.761
6	-	0.695	-	-	-	-	-	-	0.800	-	0.794
All	-	0.857	-	-	-	-	-	-	0.794	-	0.884
